# Corneal Disease & Transplantation

**DOI:** 10.3390/jcm11154432

**Published:** 2022-07-29

**Authors:** Giulia Coco, Vito Romano

**Affiliations:** 1 Department of Clinical Science and Translational Medicine, University of Rome Tor Vergata, 00133 Rome, Italy; giuliacoco@hotmail.it; 2 St. Paul’s Eye Unit, Department of Corneal Diseases, Royal Liverpool University Hospital, Liverpool L7 8XP, UK; 3 Department of Eye and Vision Science, University of Liverpool, Liverpool L7 8TX, UK; 4 Department of Medical and Surgical Specialties, Radiological Sciences, and Public Health, Eye Clinic, University of Brescia, 25121 Brescia, Italy; 5 ASST Spedali Civili di Brescia, 25123 Brescia, Italy

Corneal diseases represent the third leading cause of blindness worldwide, and corneal transplantation, which aims at restoring corneal clarity and vision, is the most frequently performed transplant worldwide. Different corneal transplantation techniques have developed over the years, from full-thickness to lamellar grafts.

One of the most important aspects of a successful corneal transplantation is effective corneal suturing, which should not only prevent complications such as wound leak, excessive scarring and infections, but also achieve a good postoperative refractive outcome [1]. Knowledge of basic mechanisms of wound healing can help in understanding the process of corneal barrier restoration [2]. Careful instrument selection might help reduce tissue stress during suture apposition [1]. Appropriate suture length and depth can reduce corneal inflammatory reaction, reduce the risk of infection, provide optimal tissue apposition and reduce postoperative astigmatism [1,3,4]. In addition, the type of suture placement can result in different patterns of tension within a three-dimensional field with different effects on corneal astigmatism [5]. Suture-related complications and their postoperative management should be known in depth, as well as specific differences that apply to pediatric patients [6,7,8,9].

Over the past few years, customized graft profiles have developed thanks to the introduction of the femtosecond laser-assisted keratoplasty, and these can help with increasing the donor–recipient apposition area with possible enhancement of wound stability and reduction of dehiscence rate [10,11].

After corneal transplantation, a visually significant complication that can occur even years after initial surgery is the post-penetrating keratoplasty corneal ectasia [12]. Gradual corneal protrusion with thinning and steepening, which determines increased irregular astigmatism, myopic shift and corneal aberrations, determines progressive visual loss [13]. Frequency, risk factors, mechanisms, diagnostic strategies, preventive measures and management can vary according to specific situations, and a comprehensive evaluation of all these features can help in deciding the best treatment option for this condition [12].

With regard to corneal diseases, keratoconus is the most common primary corneal ectasia [14]. Corneal Collagen Crosslinking (CXL) can increase corneal stiffness and, to date, is the only non-surgical treatment aimed to slow down keratoconus progression [15], and there are open questions about the definition of its progression and corneal biomechanics evaluation [16,17,18]. Long term follow-up after CXL is essential to establish its role over years in reducing keratoconus progression and eventually improving corneal astigmatism, aberrations and vision, especially if associated to transepithelial ablation [19,20].

Finally, the availability of donor tissues is limited worldwide, either because of a shortage of donors [21] or due to the inability to adequately preserve tissues in some regions. Studies aimed at evaluating more feasible ways to preserve available tissues can help by addressing the second point [22,23,24,25]. Human amniotic membrane (hAM) has vast applications in ophthalmology, and cryopreservation is the most commonly used method for its storage [26,27]. However, this requires facilities with −80 °C freezers, which are expensive both to buy and to maintain, thus limiting the use of the theoretically cost-effective hAM in developing countries. Therefore, ways to overcome this issue while maintaining appropriate biological and mechanical properties of the tissue must be explored [26].

In this issue, we aimed to highlight various aspects of corneal transplantation, corneal diseases and tissue preservation, and we hope it will be appreciated by readers.
